# A call for greater conceptual clarity in the field of mental health and psychosocial support in humanitarian settings

**DOI:** 10.1017/S2045796020001110

**Published:** 2021-01-08

**Authors:** K. E. Miller, M. J. D. Jordans, W. A. Tol, A. Galappatti

**Affiliations:** 1 War Child Holland, Amsterdam, The Netherlands; 2 Amsterdam Institute of Social Science Research, University of Amsterdam, Amsterdam, The Netherlands; 3 Department of Public Health, University of Copenhagen, Copenhagen, Denmark; 4 MHPSS.net, Batticaloa, Sri Lanka

**Keywords:** Armed conflict, framework, humanitarian, mental health, MHPSS, refugees

## Abstract

**Aims:**

When the Interagency Standing Committee (IASC) adopted the composite term mental health and psychosocial support (MHPSS) and published its guidelines for MHPSS in emergency settings in 2007, it aimed to build consensus and strengthen coordination among relevant humanitarian actors. The term MHPSS offered an inclusive tent by welcoming the different terminologies, explanatory models and intervention methods of diverse actors across several humanitarian sectors (e.g., health, protection, education, nutrition). Since its introduction, the term has become well-established within the global humanitarian system. However, it has also been critiqued for papering over substantive differences in the intervention priorities and conceptual frameworks that inform the wide range of interventions described as MHPSS. Our aims are to clarify those conceptual frameworks, to argue for their essential complementarity and to illustrate the perils of failing to adequately consider the causal models and theories of change that underlie our interventions.

**Methods:**

We describe the historical backdrop against which the term MHPSS and the IASC guidelines were developed, as well as their impact on improving relations and coordination among different aid sectors. We consider the conceptual fuzziness in the field of MHPSS and the lack of clear articulation of the different conceptual frameworks that guide interventions. We describe the explanatory models and intervention approaches of two primary frameworks within MHPSS, which we label *clinical* and *social-environmental*. Using the examples of intimate partner violence and compromised parenting in humanitarian settings, we illustrate the complementarity of these two frameworks, as well as the challenges that can arise when either framework is inappropriately applied.

**Results:**

Clinical interventions prioritise the role of intrapersonal variables, biological and/or psychological, as mediators of change in the treatment of distress. Social-environmental interventions emphasise the role of social determinants of distress and target factors in the social and material environments in order to lower distress and increase resilience in the face of adversity. Both approaches play a critical role in humanitarian settings; however, the rationale for adopting one or the other approach is commonly insufficiently articulated and should be based on a thorough assessment of causal processes at multiple levels of the social ecology.

**Conclusions:**

Greater attention to the ‘why’ of our intervention choices and more explicit articulation of the causal models and theories of change that underlie those decisions (i.e., the ‘how’), may strengthen intervention effects and minimise the risk of applying the inappropriate framework and actions to a particular problem.

When the Interagency Standing Committee (IASC) adopted the term mental health and psychosocial support (MHPSS) in 2007, it aimed to bridge a contentious divide between advocates of substantively different frameworks for addressing mental health and psychosocial needs in humanitarian settings (Inter Agency Standing Committee, 2007). The composite term MHPSS was broadly defined to include ‘… any type of local or outside support that aims to protect or promote psychosocial well-being and/or prevent or treat mental disorder’ (IASC, 2007: 1). With this expansive definition, the IASC created a large and inclusive tent by welcoming the different terminologies, explanatory models and intervention methods of those working in different sectors.

Broadly, actors within the health sector spoke of *mental health* and focused on the biological and psychotherapeutic treatment of psychiatric symptomatology, with a predominant focus on psychological trauma (Mollica *et al*., 1999; Papageorgiou *et al*., 2000; Onyut *et al*., 2005). For the purpose of this paper, we label these efforts as ‘clinical’. Those in other sectors such as protection, social welfare, education and community development generally preferred the term *psychosocial wellbeing*; they focused on strengthening resilience through preventive and promotive interventions, and on ameliorating suffering by targeting social determinants of distress such as discrimination, poverty and a lack of social support stemming from the loss or depletion of social networks (Boothby, 1992; Loughry and Eyber, 2003; Wessells and Monteiro, 2004; Betancourt and Williams, 2008). In this paper, we label these efforts as ‘social-environmental’. Obviously, this description simplifies a messier reality, where some actors adopted hybrid approaches or took positions at odds with others in their sector.

A detailed discussion of the reasons for the polarised arguments between advocates of clinical and social-environmental approaches is beyond the scope of this paper. Briefly, however, advocates of clinical interventions decried what they saw as the devaluing of much-needed treatments for highly distressed individuals suffering from the enduring effects of armed conflict, in favour of social-environmental interventions that had vaguely defined outcomes and lacked empirical evidence of impact (Yule, 2008; Neuner, 2010). Conversely, proponents of social and community-level interventions were critical of what they regarded as an overly narrow focus on posttraumatic stress disorder (PTSD), the pathologising of normal stress responses, a failure to consider the impact of ongoing environmental stressors and the medicalisation of social concerns (i.e., change-the-person solutions to problems rooted in setting-level variables that could be targeted for a change) (Loughry and Eyber, 2003; Betancourt and Williams, 2008).

In our opinion, there is robust evidence to support the legitimacy and complementarity of these substantively different frameworks, as well as merit to their respective critiques (Wessells and Van Ommeren, 2008; Ventevogel, 2018). Distress in conflict-affected communities is the result of at least two sets of causal factors: past experiences of war-related violence and loss (e.g., witnessing bomb blasts and killings, having been tortured, conflict-related sexual violence), and the constellation of ongoing stressors in protracted humanitarian settings and post-conflict situations (e.g., intimate partner violence (IPV), chronic poverty, lack of access to education and health services) (Miller and Rasmussen, 2010; Roberts and Browne, 2011; Hou *et al*., 2020). PTSD and other expressions of distress related to the violence of armed conflict are commonly identified across a range of socio-cultural settings, with levels significantly higher than in populations not affected by organised violence (Charlson *et al*., 2019), albeit less widespread than suggested by many early reports (Mollica *et al*., 1999; Neuner *et al*., 2004). Conversely, chronic, ongoing stressors generated or exacerbated by armed conflict consistently account for greater variance in mental health status than past exposure to armed conflicts and wars (Miller and Rasmussen, 2014); however, their powerful impact on mental health does not negate the reality of persistent psychological trauma and other expressions of distress stemming from past experiences of war-related violence and loss. Such evidence indicates that clinical and social-environmental approaches are by no means incompatible; on the contrary, they address different elements of interlocking concerns and hold great potential for synergy when integrated into multilevel, multi-sectoral interventions.

The essential complementarity and potential synergy of clinical and social-environmental frameworks are reflected in the IASC guidelines for MHPSS in emergency settings, the document in which the composite term MHPSS was formally introduced (IASC, 2007). The guidelines recognise the importance of a wide range of intervention strategies, from community strengthening to the specialised treatment of severe distress. They also underscore the need for multi-level programming and strong coordination among different sectors. As Tol *et al*. (2015b: 2) have noted, ‘these guidelines broadly succeeded in uniting stakeholders from very distinct theoretical and implementation approaches together under this broad shared framework for intervention’. Not everyone felt equally valued under the new guidelines, which relegated trauma treatment to a smaller and more specialised role than it had previously enjoyed (e.g., Yule, 2008). However, there was generally agreement that the guidelines represented a constructive pathway towards mutual respect and improved coordination among stakeholders in diverse sectors (Wessells and van Ommeren, 2008; Tol *et al*., 2015b; Ventevogel, 2018). In a similar vein, Ventevogel (2018: 156) has argued that the inclusive term MHPSS ‘has proven to be an important construct in bringing together professionals who work from diverse and often competing paradigms’.

Although the IASC guidelines recognised the legitimacy of different theoretical frameworks and modes of intervention, they have also been critiqued for papering over substantive theoretical and practical disagreements about how to best understand and address mental health and psychosocial needs in conflict-affected populations (Tol *et al*., 2015b; Ventevogel, 2018). Tensions between proponents of the various perspectives may have abated, but the substantive disagreements have by no means been resolved (Neuner, 2010; Miller and Rasmussen, 2014; Hynie, 2018). While the term MHPSS may have quieted the debate over whether to speak in terms of mental health treatment *or* psychosocial support, it may have also inadvertently contributed to the persistence of a conceptual fuzziness in the field (e.g., by not confronting areas of overlapping concern between frameworks, but relegating different actors into the different layers of the IASC MHPSS pyramid) (Tol *et al*., 2015b). In evaluations of MHPSS interventions reported in peer-reviewed journals, the different conceptual frameworks included under the umbrella of MHPSS are seldom explicitly articulated and it appears that interventions are often implemented without a clear theory of change or underlying causal model (Tol *et al*., 2015b; Jordans *et al*., 2016).

We suggest that a clear articulation of the core assumptions and intervention priorities of clinical and social-environmental frameworks may help to clarify the underlying causal models and hypothesised mechanisms of change that guide the development and selection of mental health and psychosocial interventions in humanitarian settings. This may in turn help clarify the rationale for the selection of specific intervention strategies. It is by now well-established that the presence of psychological distress does not in itself indicate a need for outside support, nor does it suggest the type of assistance that might be most helpful (Norris *et al*., 2002; Bonanno, 2004; Silove and Steel, 2006). Only a careful assessment of those factors giving rise to and maintaining distress can provide a clear rationale for the selection of intervention strategies.

Rather than seeking to divide humanitarian practitioners into different ‘camps’, we hope that a clearer articulation of hypothesised theories of change will assist in improved understanding between diverse humanitarian actors in relation to the complementarity of their efforts. With a clearer understanding of why and how MHPSS interventions are deployed, can come a clearer understanding of where there are overlapping interests and potential synergies.

With regard to monitoring and evaluation, clarifying our causal models and theories of change can also help ensure that specified outcomes correspond to stated intervention goals and processes. There is still a tendency, for example, for evaluations of preventive and promotive interventions to prioritise the measurement of clinical outcomes that are inconsistent with a focus on prevention and the promotion of psychosocial wellbeing (Purgato *et al*., 2018; Haroz *et al*., 2020). In their review of life skills interventions for adolescents in low and middle-income countries (LMICs), for example, Singla *et al*. (2019) found that a reduction in symptoms of PTSD and depression symptoms were frequently designated as primary outcomes, rather than a strengthening of specific life skills such as social problem-solving, emotion regulation and strengthening relationships with parents.

Finally, greater conceptual clarity may also help avoid the inappropriate application of a particular framework to a specific problem. This can occur, for example, when suffering that is primarily the result of ongoing family violence is misattributed to previously experienced war trauma and treated psychotherapeutically, rather than by first working to establish safety and addressing the use and roots of violence within the family. This can be avoided by assessing the factors contributing to distress both temporally (past and present) and at multiple levels of the social ecology.

Our purpose is *not* to rekindle or exacerbate the debate over which conceptual framework holds a greater claim to truth; on the contrary, we argue for multilevel interventions that integrate diverse perspectives that are grounded in an analysis of causal factors both historical and current, and at multiple ecological levels. Our hope is to draw attention to the importance of achieving greater clarity regarding the ‘why’ and ‘how’ of our intervention choices and to encourage more explicit articulation of the theories of change that underlie those decisions.

## Conceptual frameworks in the field of MHPSS

The major conceptual frameworks within the field of MHPSS have been described in various ways: *biomedical, biological, psychological, trauma-focused, public health, holistic* and *psychosocial* are among the more commonly used labels (Galappatti, 2003; Wessells and van Ommeren, 2008; Miller and Rasmussen, 2010; Jordans *et al*., 2016; Ventevogel, 2018). However, we would like to posit two primary conceptual frameworks underlying much of the variation amongst these approaches, which we refer to in this paper as clinical and social-environmental.

### Clinical

The clinical framework actually comprises two underlying frameworks that differ in significant ways, but which share an emphasis on the treatment of severe distress and impairment by targeting intrapersonal factors as mediators of change.

One of these underlying frameworks is *psychological*. Interventions resulting from a predominantly psychological theory of change are commonly psychotherapeutic and are usually aimed at altering psychological variables such as emotional dysregulation, maladaptive cognitions, dysfunctional behaviour patterns and a failure to adequately process trauma-related memories and affects. Psychological interventions in conflict-affected communities were historically focused on the assessment and treatment of PSTD, which was widely assumed to be the most important result of prior exposure to war-related violence and loss (Mollica *et al*., 1999; Neuner *et al*., 2004; Nicholl and Thompson, 2004). However, this emphasis on war trauma has gradually given way to a broader view, in which severe distress is understood to result from a wide variety of painful life events beyond direct exposure to armed conflict. Psychological interventions have traditionally been implemented by mental health professionals; however, there is a growing body of evidence supporting their effectiveness when implemented by trained and supervised non-specialists (van Ginneken *et al*., 2013), and ‘low-intensity psychological interventions’ are increasingly being developed for non-specialist delivery (de Graaf *et al*., 2020; Tol *et al*., 2020). The agent of delivery (e.g., a psychotherapist or counsellor) does not determine their framing as psychological; rather, it is the focus of their theory of change on psychological variables as mediators of change.

The other underlying approach within the clinical framework is *biological*. Similar to psychological interventions, biological interventions in humanitarian settings are generally used in the treatment or management of severe distress; however, they have a broader application, which includes the treatment of individuals with serious mental and neurological illnesses not necessarily related to humanitarian crises, such as psychosis and epilepsy. Within the biological framework, there is a recognition of the root causes of suffering in violence, displacement and poverty, among other factors; however, the focus has increasingly shifted to proximal factors of a psychophysiological nature that are believed to contribute to the persistence of distress and dysfunction. Interventions are consequently primarily pharmacological and include a spectrum of medications meant to alleviate or manage distress and improve functioning. Thus, like psychological interventions, their focus is on changing intrapersonal variables to improve mental health.

Not much is known about prescribing practices in humanitarian settings, because the research literature has tended to focus more on the evaluation of psychological interventions. This is evident in the overwhelming preponderance of research on psychological interventions relative to that on biological treatments in war-affected communities in LMICs (Jordans *et al*., 2016; O'Sullivan *et al*., 2016; Bangpan *et al*., 2017; Purgato *et al*., 2018; Barbui *et al*., 2020).

We also note that while psychotherapeutic interventions predominate within the clinical framework, psychological methods may also be used in non-clinical interventions aimed at fostering resilience by strengthening intrapersonal resources such as self-esteem and the capacity for stress management (Papola *et al*., 2020). It is preferable to reduce or eliminate noxious stressors than it is to require that people adapt to harmful environments; however, in settings where persistent stressors cannot readily be altered, psychological interventions aimed at strengthening resilience can play an important role. Emerging research that identifies complex, bi-directional relationships between exposure to adversity and psychological distress, indicates the importance of interventions that combine efforts aimed at addressing social determinants and intra-personal psychological processes (Tol, 2020).

### Social-environmental

The second framework is *social-environmental* in nature. As described earlier, theories of change with a social-environmental starting point prioritise the role of setting-level variables in causing and maintaining distress – factors such as overcrowded and unsafe housing, poverty, unemployment, social exclusion and family violence that may be caused or exacerbated by chronic stress (Fernando *et al*., 2010; Betancourt *et al*., 2013; Tol *et al*., 2013; Hynie, 2018). Social-environmental interventions span a broad gamut of methodologies but share a focus on altering settings and mobilising social resources to lower distress, strengthen wellbeing and foster resilience (Loughry and Eyber, 2003; IASC, 2007; UNHCR, 2013; Haroz *et al*., 2020). Actors working within a social-environmental framework may also prioritise social outcomes (e.g., repairing the damage of armed conflicts on the social and moral fabric of communities) as primary outcomes important in and of themselves, not just as mediators on the way to reducing adverse psychological states.

It may be noted that we have not included a *psychosocial* framework in this paper, despite the ubiquity of the term psychosocial in the MHPSS literature. This decision reflects our wish to avoid the confusion arising from (at least) two very different understandings of the term psychosocial within the MHPSS community. In the health sector, the term psychosocial usually refers to all non-pharmacological interventions aimed at alleviating distress and disorder and improving functioning (Institute of Medicine, 2015; Barbui *et al*., 2020). In this view, psychological and social-environmental approaches are grouped together based on what they are *not* (pharmacological), despite their different explanatory models, intervention methods and prioritised outcomes. Outside of the health sector, in contrast, the term *psychosocial* is often roughly analogous to what we are calling *social-environmental*. Thus, psychosocial approaches emphasise the role of setting-level variables, such as safety and support within the family and community, lack of access to income generation and educational opportunities and the adequacy of housing and other material resources, as these affect mental health and psychosocial wellbeing.

We have no interest in advocating for one or the other definition of psychosocial, perhaps an important discussion but one which lies beyond the scope of this paper. We do note with concern, however, the confusion that may arise when such a widely used term is employed differently by different actors all working to strengthen the mental health of conflict-affected populations. We hope the term *social-environmental* helps side-step this confusion and reflects clearly the setting-level focus of this explanatory and intervention framework.

## The link between conceptual frameworks and effective interventions

Nearly 50 years ago, Caplan and Nelson observed that how we explain a problem generally determines how we go about trying to solve it (Caplan and Nelson, 1973). Thus. psychological explanations of distress typically give rise to psychological interventions, just as social-environmental analyses generally lead to interventions aimed at altering aspects of the social or material environment. For example, if we believe that symptoms of PTSD reflect the enduring impact of prior trauma exposure, we are likely to focus on various intrapersonal processes (e.g., impaired emotion regulation, overgeneralised threat perception, shame and self-blame) using any of a variety of evidence-based psychotherapeutic treatments. This approach has been shown to be helpful in addressing the post-traumatic effects of sexual assault, torture and other forms of traumatic stress that are tragically common in settings of organised violence (Weiss *et al*., 2015; Bangpan *et al*., 2017; Nosè *et al*., 2017).

Alternatively, if we believe that PTSD symptoms reflect the effects of ongoing IPV, we are more likely to intervene by trying to stop the abuse, for example through mediation, court interventions, or by helping the victim find safety outside of the home. If IPV is also a product of widespread normalisation of violence against women within the community, a social-environmental intervention that aims to change social attitudes may also be indicated. Psychological interventions may also be indicated, for example, in supporting a victim to explore means of harm reduction or possible actions to protect herself. Moreover, if psychological problems contribute to the perpetration of IPV by partners (e.g., alcohol misuse or psychological distress resulting from partners' exposure to political violence), clinical interventions may be employed simultaneously with protective interventions. And if trauma symptoms persist after safety has been established, psychological treatment may then be indicated in order to restore emotional wellbeing. These multi-pronged approaches illustrate the sort of complementarity envisioned in the IASC guidelines.

Finally, trauma symptoms may be multiply determined, with etiological roots in previous events as well as ongoing circumstances. A survivor of sexual assault may well be traumatised by her terrifying experience, but her distress may also reflect a realistic fear of social rejection by her family and community (Kelly, 2011). Similarly, depending on the cultural context, her hyperarousal may stem from a very real danger of being killed by relatives in order to restore her family's honour (Husseini, 2009). Addressing concerns around social rejection and safety are likely to be prerequisites for healing the trauma of the actual sexual violence. Here again, we see the value of integrating social-environmental and clinical perspectives.

It is a truism that specifying the theoretical and causal mechanisms for any mental health intervention is an important step to achieving and demonstrating impact. Doing so forces us to articulate our understanding of those factors that we believe underlie or contribute meaningfully to the problems we wish to address. The specification of these variables allows us to develop a theory of change that explains and justifies our intervention methodology or treatment plan (Breuer *et al*., 2016). To be clear, the existence of distress does not, by itself, indicate what type of support might be most helpful. Only a thorough assessment of causal or contributing factors can indicate which approaches may serve to intervene most effectively.

Interventions or treatments that are ineffective may simply reflect assessments that fail to adequately specify critical sources of distress and key mediators of change. This can easily happen in work with conflict-affected communities where complexity (e.g., exposure to multiple stressors, both past and current) is the norm. Miller (2016) has illustrated this with the example of a Bosnian woman with severe PTSD who was being treated at a refugee clinic in the USA. The clinic staff attributed her trauma symptoms to the terrible violence she had witnessed before leaving Bosnia, and her treatment, therefore, focused on resolving her persistent war trauma. After months during which neither counselling nor psycho-pharmacology proved helpful, it emerged that she was being repeatedly sexually assaulted by her husband in their small one-room apartment, in the presence of their traumatised young child. The salience of her war experience had led the clinical team to overlook the possibility that current trauma might explain her extreme and persistent distress. Once the abuse was discovered, a fundamentally different intervention strategy was developed, focused on getting her and her child out of the home and into the safety of a shelter. Once there, they both received counselling aimed at helping them heal from the effects of the abuse they had endured. Again, we see the temporal complementarity of clinical and social-environmental frameworks. In this case, however, a more thorough assessment of current environmental stressors might have prevented months of suffering.

As noted, one of the central challenges in developing theories of change for interventions in conflict-affected communities is the diversity of ongoing stressors to which people are exposed, combined with their history of exposure to violence and loss. Moreover, as we illustrated in the previous example, the striking nature of the violence from which people have escaped (or through which they may still be living in settings of ongoing conflict), can easily overshadow less obvious yet equally powerful and more immediate sources of distress. Recent research has built on a small number of earlier studies (e.g., Punamäki, 1990; Al-Krenawi *et al*., 2007) to establish the significant role of compromised parenting in explaining distress among children exposed to armed conflict. Harsh, abusive and unresponsive parenting have been found to powerfully mediate the relationship of armed conflict and forced migration to children's mental health (Palosaari *et al*., 2013; Sim *et al*., 2018; Eltanamly *et al*., 2019). Persistently high levels of stress and distress can undermine the best intentions of parents, whose own needs for emotional and practical support often go unaddressed in humanitarian settings (Miller *et al*., 2020a). The robust evidence regarding the impact of chronic stress on parenting and in turn on children's mental health, has gradually begun to expand intervention efforts beyond the traditional focus on direct work with children to include efforts at strengthening the wellbeing and parenting of their primary caregivers (Jordans *et al*., 2013; Puffer *et al*., 2017; Stark *et al*., 2018; Miller *et al*., 2020b). It has become increasingly clear that it makes little sense to treat distressed children only to send them home to family dynamics that may be perpetuating their distress. In fact, the historical neglect of parenting and parental wellbeing in humanitarian settings may help explain why both treatment-focused and preventive interventions with conflict-affected children have generally shown modest and inconsistent effects (Jordans *et al*., 2016; Bangpan *et al*., 2017; Purgato *et al*., 2018).

A similarly broadened perspective can be seen regarding IPV, which until recently was seldom assessed in research on mental health in conflict-affected populations (Clark *et al*., 2010; Rubenstein and Stark, 2017; Rubenstein *et al*., 2020; Tol, 2020). Although IPV has long been a central topic of concern within the gender-based violence community, researchers focused specifically on armed conflict and mental health have historically neglected to ask about violence within the home (Rubenstein and Stark, 2017). As researchers expanded their focus beyond the effects of direct exposure to armed conflict, however, they began to document the deleterious effects of ongoing environmental stressors, including IPV. There is now a growing body of evidence which suggests that one key pathway by which political violence affects women's mental health is through its impact on their male partners, whose risk of becoming violent at home has been shown to increase following experiences of violence and humiliation at the hands of police, soldiers or other armed combatants (Clark *et al*., 2010; Khamis, 2016; Rees *et al*., 2018). It has become clear that psychological distress among women in communities affected by organised violence may reflect the impact of (traumatic) stressors both past and present, beyond and within the home. This expanded view implies the need for an integrated, multi-sectoral, ecologically diverse range of intervention strategies to address IPV in conflict-affected communities. This includes, for example, programs aimed at both preventing and reducing the use of violence by men against their partners, the provision of safe refuge for women and children when violence cannot be stopped and the treatment of distress among survivors of IPV, including children who have witnessed violence against their mothers. This again reflects precisely the sort of complementarity envisioned by the IASC in its 2007 guidelines.

## Conclusion

We suggest that clarifying the distinction between clinical (biological and psychological) and social-environmental frameworks may facilitate the development of sound theories of change by drawing our attention to both intrapersonal and environmental factors and processes, past and present, that affect mental health and wellbeing in humanitarian settings. This specification may in turn help ensure that our interventions reflect an appropriately multilevel analysis of causal factors and putative mediators of change. In this way, we can decrease the risk of overlooking key social determinants that might be contributing to distress, or conversely, of failing to consider the impact of prior trauma on mental health in settings where current stressors are especially salient. Stated differently, we believe that greater conceptual clarity can help researchers and practitioners better articulate the underlying mechanisms of the interventions they employ and the rationale for their intervention strategies.

This paper is also a call for multilevel interventions, and indeed, the available evidence on factors affecting mental health in humanitarian settings points in precisely this direction. However, despite repeated calls for interventions that target mediators of change at multiple levels of the social ecology, to date, few such ecological interventions are found in the literature. Numerous reviews reflect the same persistent reality: clinical and social-environmental interventions are seldom integrated into multilevel, integrated systems of support (Barenbaum *et al*., 2005; Ellis *et al*., 2012; Betancourt *et al*., 2013; Jordans *et al*., 2016). For organizations and institutions wishing to maximise their impact, we advocate a systems perspective on provision of care in which ecologically diverse interventions are well-coordinated and closely linked (Jordans *et al*., 2010; Ellis *et al*., 2012; Jordans *et al*., 2018).

### Other conceptual frameworks

In this paper, we have focused on what we regard as the two primary conceptual frameworks, clinical and social-environmental, that guide the development and selection of interventions in conflict-affected communities. However, we recognise that other conceptual frameworks inform the way mental health and psychosocial wellbeing are addressed in humanitarian settings. For example, spiritual beliefs and practices often play a powerful role in supporting healing and strengthening resilience in the wake of violence, displacement and natural disaster (Galappatti, 2003; Ager *et al*., 2015; Miller, 2016), yet spiritual frameworks for understanding and addressing suffering in humanitarian contexts have received little empirical attention to date (Ager *et al*., 2015). The same is true with the *social justice* framework advocated by Tol (2020), whose recent articulation of this framework extends the earlier work of psychologists working in settings of extreme inequality and systematic oppression, including apartheid-era South Africa (Swartz *et al.*, 1990) and Central America (Martín-Baró, 1989). Such frameworks evoke dimensions of suffering and wellbeing that our paper has not engaged with. In focusing our discussion on the clinical and social-environmental frameworks that currently predominate, our aim was not to minimise the importance of other approaches such as these to explaining and reducing suffering in the wake of violence and displacement. On the contrary, we believe strengthening the evidence base for these and possibly other conceptual frameworks can only benefit communities living in adversity.

### The map is not the territory: conceptual frameworks and real-world complexities

Conceptual frameworks are always rough approximations of real-world phenomena (Korzybski, 1931). We recognise that interventions aimed at improving mental health and wellbeing in humanitarian settings may not fit neatly into the categories delineated in this paper. For example, sociotherapy, as implemented in post-genocide Rwanda is a group intervention focused simultaneously on healing trauma among individual participants while fostering reconciliation and repairing the social fabric torn apart by the genocide (Richters *et al*., 2008; Jansen *et al*., 2015).

Nonetheless, the frameworks we have described do correspond reasonably well to prevailing approaches to understanding and addressing the mental health and psychosocial needs of conflict-affected communities. More importantly, these frameworks are heuristically useful because they encourage us to be explicit about the causal models and theories of change that guide our interventions, drawing our attention to putative causal factors and potential targets of change at different ecological levels.

It is certainly possible to maintain a distinction between conceptual frameworks and to better articulate causal models and theories of change while continuing to embrace the umbrella term MHPSS. Indeed, we are not suggesting that the term should be abandoned; on the contrary, it has served an important unifying function among individuals and organisations working from diverse perspectives to strengthen mental health in conflict-affected populations (Ventevogel, 2018). Rather, we aim to refocus attention on the different frameworks included within this umbrella term, to illustrate their unique explanatory models and intervention strategies, and to underscore their fundamental complementarity and potential for integration in multisectoral interventions.
